# Type Iγ phosphatidylinositol phosphate kinase regulates PD-L1 expression by activating NF-κB

**DOI:** 10.18632/oncotarget.17123

**Published:** 2017-04-15

**Authors:** Junli Xue, Chunhua Chen, Manlong Qi, Yan Huang, Lin Wang, Yong Gao, Haidong Dong, Kun Ling

**Affiliations:** ^1^ Department of Oncology, Shanghai East Hospital, Tongji University School of Medicine, Shanghai 200120, China; ^2^ Department of Biochemistry and Molecular Biology, Mayo Clinic, Rochester, MN 55902, USA; ^3^ Department of Clinical Genetics, Shengjing Hospital of China Medical University, Shenyang 110004, Liaoning Province, China; ^4^ Department of Oncology, Guangzhou Red Cross Hospital, Guangzhou 510220, Guangdong Province, China; ^5^ Department of Urology, Mayo Clinic, Rochester, MN 55902, USA

**Keywords:** PD-L1, triple negative breast cancer, PIPKIγ, NF-κB, AKT

## Abstract

The programmed death-ligand 1 (PD-L1), by binding to PD-1 on the surface of immune cells, activates a major immune checkpoint pathway. Elevated expression of PD-L1 in tumor cells mediates tumor-induced T-cell exhaustion and immune suppression; therefore protect the survival of tumor cells. Although blockade of the PD-1/PD-L1 axis exhibits great potential in cancer treatment, mechanisms driving the up-regulation of PD-L1 in tumor cells remain not fully understood. Here we found that type Iγ phosphatidylinositol 4-phosphate (PtdIns(4)P) 5-kinase (PIPKIγ) is required for PD-L1 expression in triple negative breast cancer cells. Depletion of PIPKIγ inhibits both intrinsic and induced PD-L1 expression. Results from further analyses suggest that PIPKIγ promotes the transcription of the PD-L1 gene by activating the NF-κB pathway in these cells. These results demonstrate that PIPKIγ-dependent expression of PD-L1 is likely important for the progression of triple negative breast cancer.

## INTRODUCTION

It has been well established that cancer cells often express variant tumor-specific antigens that can be recognized by the immune system [1–2], which leads to spontaneous anti-tumor immune response. However, cancer cells can manage to evade the destruction by reducing the immunogenicity and/or inducing immunosuppression [3]. For example, the signaling axis of programmed death-1 (PD-1) and its ligand PD-L1, one of immune checkpoint pathways, is frequently hijacked by cancer cells to achieve immune escape [2, 4, 5]. Many types of cancer cells up-regulate the expression of PD-L1, which binds to PD-1 expressing on the surface of T-cells and induces the programmed death in activated T-cells [1, 6, 7–10]. Indeed, the upregulation of PD-L1 has been correlated with poor prognosis of variant types of malignancies [11]. At present, immune checkpoint blockade is extensively adopted by tumor researchers and clinicians [5]. Results from recent clinical trials demonstrate that patients responding to anti-PD therapies benefit from a sustainable, complete tumor repression on multiple types of hematological and solid malignancies including breast cancer [12]. However, the responsive rate and efficacies are not ideal. Understanding the mechanism regulating the expression of PD-L1 in cancer cells may help researchers to define better strategies for efficacious immunotherapy targeting the PD pathway.

Abundant studies have been developed to evaluate PD-L1 in breast cancer patients. Current literature suggests that PD-L1 is often upregulated in basal-like, triple negative breast cancer (TNBC). It is also shown that tumors highly expressing PD-L1 tend to bear more tumor-infiltrating lymphocytes (TIL) than the PD-L1 negative or low tumors, which reflects stronger local immune response against tumors. However, the prognostic role of PD-L1 in breast cancer is quite controversial. In different studies, expression of PD-L1 was observed correlating with better prognosis [13–17], with poor prognosis [18–23], or having no correlation with the outcomes [24]. A meta-analysis employed 5 studies involving 2061 breast cancer patients revealed that the high level of PD-L1 correlates with poor prognosis, high lymph node metastasis, poor nuclear grade and negative ER expression [25]. Nevertheless, blockade of PD-L1 binding to PD-1 has emerged as a very promising strategy in many recent clinical trials, however the detectable expression level of PD-L1 did not show correlation with the response to PD blockade. In this context, understanding the mechanism regulating the expression of PD-L1 in breast cancer is important for defining potential targets or developing novel strategies to achieve more sensitive detection of PD-L1 expression and/or more precise prediction of patient response toward anti-PD therapies.

The type I phosphatidylinositol 4-phosphate 5-kinases (PIPKIs) are a family of enzymes that synthesize phosphatidylinositol 4,5-bisphosphate [PI(4,5)P_2_]. It comprises a family of lipid kinases encoded by three genes that give rise to PIPKIα, PIPKIβ and PIPKIγ. The three PIPKI isoforms (α, β and γ) share very conserved kinase domain but have a high level of sequence divergence at the C-terminus, which allows for their distinct localization and function [26]. Others and we have reported the important role of PIPKIγ in multiple cellular processes that regulate tumor progression, such as the assembly of cell-cell and cell-matrix adhesions, directional cell migration and invasion, and metastasis [27, 28]. Indeed, PIPKIγ modulates invasion and proliferation and its expression correlates with poor prognosis in breast cancer [29]. In our previous work, we revealed that PIPKIγ as downstream factor of EGF plays an important role in breast cancer progression [30]. In this study, we found that depletion of PIPKIγ inhibits the expression of PD-L1 in multiple types of TNBC cells. Our results suggest a novel mechanism that PIPKIγ is necessary for PD-L1 expression by activating the binding of NF-κB to the promoter of PD-L1. These data support a novel role of PIPKIγ in TNBC by regulating PD-L1 expression in addition to directly supporting tumor survival and metastasis. Furthermore, current study suggests an interesting possibility that PIPKIγ might be a potential drug target for breast cancer therapy and may improve the efficacy of PD-blockade therapies when combined with anti-PD reagents.

## RESULTS

### Loss of PIPKIγ causes a decreased expression of PD-L1 in TNBC cells

**Figure 1:**
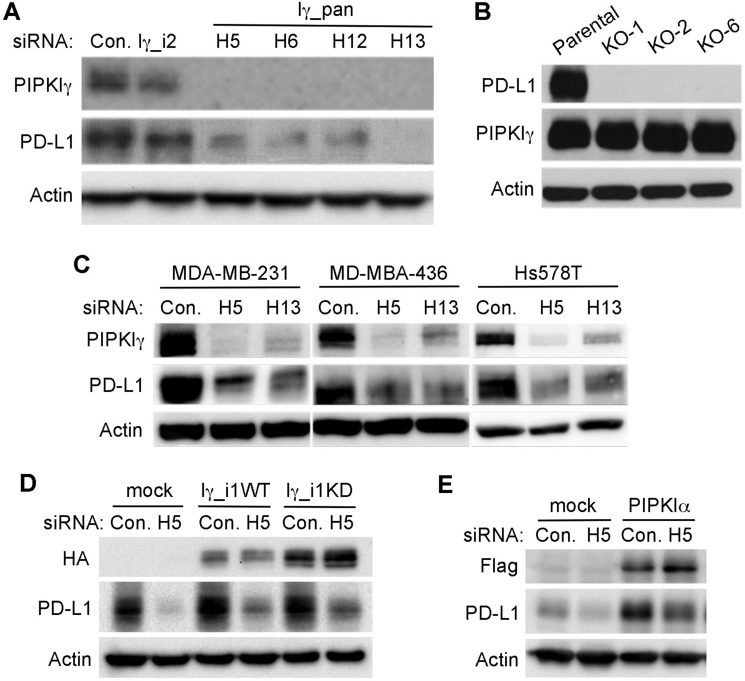
PIPKIγ-depleted TNBC cells exhibits downregulated PD-L1 expression (**A**) MDA-MB-231 cells were transfected with non-specific control (Con.), human pan-PIPKIγ (Iγ-pan, 4 distinct siRNAs including H5, H6, H12, H13), or human PIPKIγ_i2-specific (Iγ_i2) siRNAs, respectively. 48 hrs post transfections, cells were collected for immunoblotting analyses using indicated antibodies. (**B**) Three PD-L1-null MDA-MB-231 single clones (KO-1, KO-2, KO-6) were constructed using CRISPR/Cas9 system. PIPKIγ expression in the parental and individual PD-L1-null cell lines was determined by immunoblotting. (**C**) Three different human TNBC cell lines were transfected with control or pan-PIPKIγ siRNAs (H5 and H13) and then PD-L1 expression was accessed by immunoblotting. (**D**) Hs578T cells were transfected to express control empty vector (mock), H5-resistant mouse PIPKIγ_i1 wild type (Iγ_i1WT) or kinase dead (Iγ_i1KD) for 24 hrs, and then transfected with control (Con.) or HA-tagged human pan-PIPKIγ specific (H5) siRNAs for another 48 hrs. These cells were then lysed and subjected to immunoblotting with indicated antibodies. (**E**) Hs578T cells were handled as described in (**D**) except for using Flag-tagged human PIPKIα to replace HA-tagged mouse PIPKIγ_i1 construct.

Our previous work using 4T1 cells, a mouse spontaneous TNBC cell line, demonstrated that PIPKIγ is required for the progression of TNBC [31]. In this study, results from the *in vivo* orthotopic transplantation model indicated that PIPKIγ-deficient 4T1 tumors associated with an unfavorable microenvironment comparing to normal 4T1 tumors. This includes significantly decreased angiogenesis and tumor-infiltrated macrophages [31], suggesting that PIPKIγ helps TNBC cells to remodel the host environment including the immune response. Surprisingly, we recently found that loss of PIPKIγ led to a decreased expression of PD-L1 in human TNBC cells. As shown in Figure 1A, the protein levels of PD-L1 in PIPKIγ-depleted human TNBC cell line MDA-MB-231 cells were significantly deceased. We observed similar level of PD-L1 downregulation when treating these cells with four distinctive siRNAs that target all PIPKIγ isoforms (pan-PIPKIγ). However, the PIPKIγ isoform-2 (PIPKIγ_i2) specific siRNA had no effect on PD-L1 expression, indicating that it is likely PIPKIγ_i1, but not PIPKIγ_i2, regulating the levels of PD-L1 protein. To determine whether PD-L1 can affect the expression of PIPKIγ, we examined the levels of PIPKIγ in PD-L1 knockout MDA-MB-231 cell lines created using CRISPR/Cas9 system. In all three PD-L1 knockout clones, PIPKIγ protein levels maintained the same, indicating that PD-L1 has no effect on PIPKIγ expression in MDA-MB-231 cells (Figure 1B). Moreover, we found that the expression of PD-L1 in another two human TNBC cell lines, MDA-MB-436 and Hs578T, was also decreased upon PIPKIγ depletion (Figure 1C). Overexpression of RNAi-resistant wild type PIPKIγ_i1 not only increased the PD-L1 expression in control cells, but also partially restored the decreased PD-L1 expression in PIPKIγ–depleted cells (Figure 1D). Interestingly, overexpressed kinase dead PIPKIγ_i1 (Figure 1D) and wild type PIPKI*α* (Figure 1E) also improved PD-L1 expression in PIPKIγ-depleted cells, although depletion of endogenous PIPKIα had no effect on PD-L1 expression (data not shown). In the context that the kinase dead PIPKIγ retains a very low kinase activity when highly overexpressed, we reason that PtdIns(4,5)P_2_ likely promotes PD-L1 expression. Nevertheless, our data suggest a novel role of PIPKIγ in regulating PD-L1 expression in TNBC cells. In the context that upregulation of PD-L1 in TNBC cells plays a critical role in the immune evasion of these cells, these results suggest a unique mechanism and potential drug targets for inhibiting PD-L1 expression in TNBC cells.

**Figure 2:**
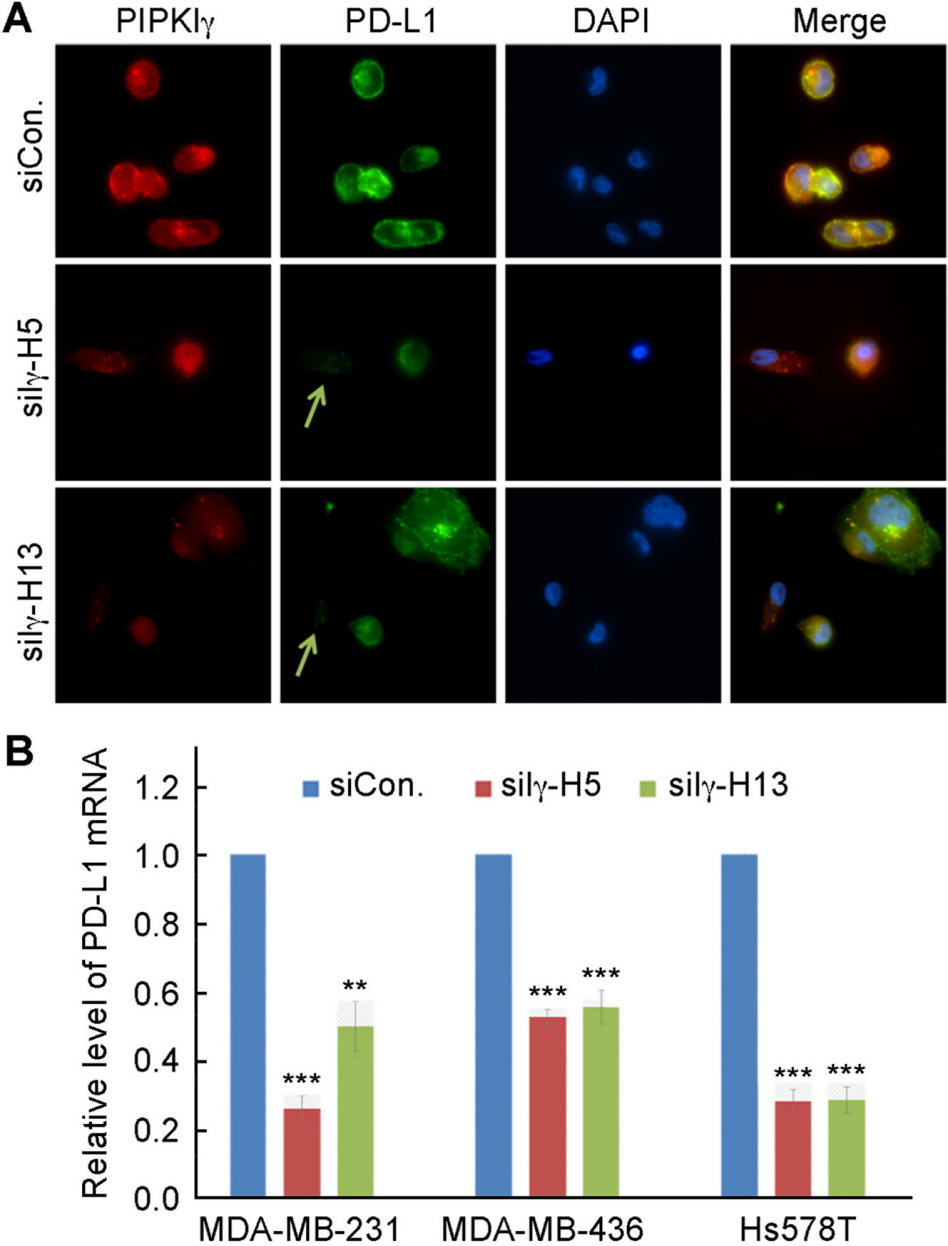
Loss of PIPKIγ inhibits PD-L1 transcription (**A**) After transfected with control (Ctrl) or pan-PIPKIγ-specific siRNAs (H5 and H13) for 48 hrs, MDA-MB-231 cells were subjected to indirect immunofluorescence with antibodies against PD-L1 (green), PIPKIγ (Iγ855, red), and DAPI (blue), and then analyzed under epi-fluorescence microscope. Arrows indicate the PIPKIγ–depleted cells where PD-L1 showed substantially decreased signal comparing to control cells expressing normal level of PIPKIγ. (**B**) Three different TNBC cell lines were transfected with control or pan-PIPKIγ siRNAs (H5 and H13) for 48 hrs. Then the levels of PD-L1 mRNA in cells of each group were accessed by quantitative RT-PCR (qRT-PCR) using specific PD-L1 primers with GAPDH as the internal control. Results from at least three independent experiments were quantified, statistically analyzed, and plotted as mean ± S.E.M.

To understand how PIPKIγ possibly regulates PD-L1 levels, we first investigated whether the subcellular localization of PD-L1 was changed when PIPKIγ was absent. Results from immunofluorescence microscopy stydies indicated that PD-L1 enriches in both the plasma membrane and certain cytoplasmic vesicular compartments (Figure 2A). In cells where PIPKIγ expression was suppressed by RNAi, PD-L1 signals were strongly diminished at both locales (Figure 2A), further confirming that loss of PIPKIγ leads to decreased PD-L1. Meanwhile, we also performed quantitative reverse transcription polymerase chain reaction (qRT-PCR) to determine whether the level of PD-L1 mRNA was affected in PIPKIγ-depleted cells. In agreement with the immunoblotting results shown in Figure 1, three TNBC cell lines all exhibited substencially decreased PD-L1 mRNA when PIPKIγ was depleted (Figure 2B). Our results suggested that PIPKIγ plausibly participates in regulating the intrinsic transcription of PD-L1 gene in TNBC cells.

### Induced PD-L1 expression in TNBC cells requires PIPKIγ

**Figure 3:**
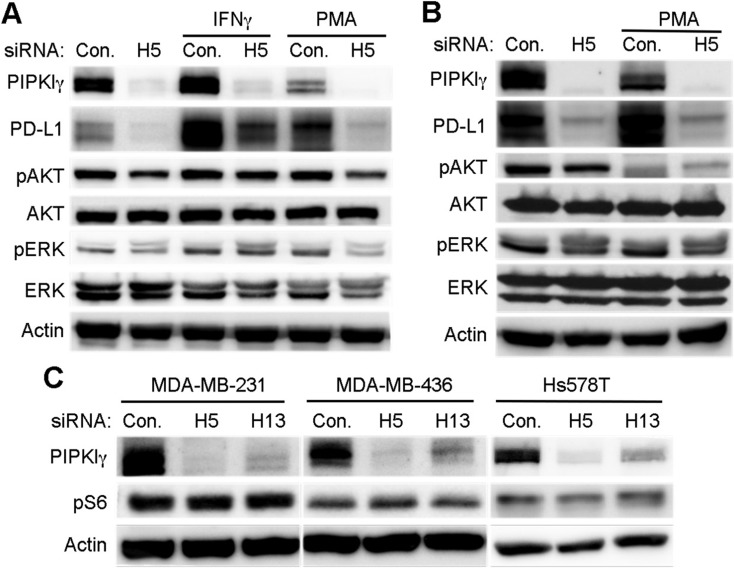
PIPKIγ regulates the induced-expression of PD-L1 in TNBC cells independent of AKT (**A**) 48 hrs after transfection with control (Ctrl) or pan-PIPKIγ (H5) siRNA, Hs578T cells were treated with 10 ng/mL PMA for 12 hrs or 30 ng/mL IFN-γ for 48 hrs. Then cells were lysed and analyzed by immunoblotting using indicated antibodies. Iγ855, pan-PIPKIγ; pAKT, phospho-AKT (pAKT); pERK (phospho-ERK). (**B**) MDA-MB-231 cells were transfected with control or pan-PIPKIγ (H5) siRNA for 48 hrs and then stimulated with 10 ng/mL PMA for 12 hrs. Cells were then subjected to immunoblotting analyses as described in A. (**C**) Three TNBC cell lines were treated with control or pan-PIPKIγ (H5 and H13) siRNAs for 48 hrs and then analyzed by immunoblotting with indicated antibodies. pS6, phosphorylated S6.

The expression of PD-L1 in tumor cells could be controlled by intrinsic or extrinsic signals. In established human breast cancer cell lines, intrinsic PD-L1 expression is only observed in some types of TNBC cells, in which PD-L1 level can be further increased by extracellular stimuli. It has been well established that IFN-γ, generated by the host immune cells, is the most potent proinflammatory cytokine that induces the extrinsic expression of PD-L1 in multiple types of tumor cells *in vivo*, therefore plays a critical role in the development of tumor immune escape [7]. In TNBC cells cultured *in vitro*, we indeed observed a significant increase of PD-L1 upon IFN-γ treatment (Figure 3A). Interestingly, depletion of PIPKIγ strongly blocked IFN-γ induced PD-L1 upregulation (Figure 3A). Activation of protein kinase C was also reported to stimulate PD-L1 expression via activating PKD in oral squamous cell carcinoma [32]. PtdIns(4,5)P_2_, the product of PIPKIγ, can be cleaved by phospholipase C to generate diacylglycerol that activates PKC and PKD, suggesting that PIPKIγ may play a role in PKC/PKD activated PD-L1 expression. In our hands, incubation of 10 ng/mL phorbol 12-myristate 13-acetate (PMA, PKC activator) for 24-hr indeed enhanced PD-L1 expression in both MDA-MB-231 and Hs578 cells (Figure 3A and 3B). Although PMA also caused a slight decrease of PIPKIγ expression, we reason that the remaining PIPKIγ is sufficient to support PD-L1 expression. Nevertheless, depletion of PIPKIγ completely inhibited PMA-induced PD-L1 expression (Figure 3A). Our results strongly suggest that in TNBC cells, PIPKIγ likely participates in the regulation of both the intrinsic and induced PD-L1 expression at multiple levels.

Current published works suggest that the intrinsic control of increased PD-L1 expression in solid tumors may result from the abnormally upregulated PI3K, MAPK, or Wnt pathways [33]. The extrinsic signals such as IFN-γ are reported to stimulate PD-L1 expression in different cell types by activating variant signaling pathways, including PI3K/AKT, RAF/MAPK, and JAK/STAT [33]. However, signaling pathways promoting PD-L1 upregulation in breast cancers especially in TNBC cells remain unclear. In our hands, neither IFN-γ nor PMA changed the activity of AKT or MAPK in MDA-MB-231 cells, although both of them stimulated PD-L1 expression (Figure 3A and 3B). In Hs578T cells, PMA inhibited the activation of AKT but left MAPK activity unchanged, although it increased PD-L1 expression (Figure 3B). These results suggested that in TNBC cells, IFN-γ or PKC-induced PD-L1 expression is independent of AKT or MAPK. Consistently, PIPKIγ-depleted cells showed similar AKT or MAPK activation as control cells responding to IFN-γ stimulation (Figure 3A), indicating that although PIPKIγ appears necessary for IFN-γ induced PD-L1 expression, it is not by impairing AKT or MAPK activation.

On the other hand, PIPKIγ depletion indeed led to a decreased AKT activation in non-treated, resting TNBC cells (Figure 3A and 3B), suggesting that PIPKIγ may participate in the intrinsic PD-L1 expression by facilitating the activation of PI3K/AKT pathway. PI3K/AKT activation, via the mTOR-mediated phosphorylation of S6 ribosomal protein (pS6), results in the upregulation of PD-L1 [34, 35]. However, we did not observe any change in pS6 levels in PIPKIγ-depleted cells comparing to control cells (Figure 3C), indicating that loss of PIPKIγ has no effect on the post-transcriptional regulation of PD-L1. Interestingly, PMA treatment that led to PD-L1 upregulation caused a significant decrease on AKT phosphorylation in Hs578T cells (Figure 3B), indicating that PKC activation stimulates PD-L1 expression via an AKT-independent pathway. Therefore, our data suggest that PIPKIγ has no effect on the post-transcriptional regulation of PD-L1 via the AKT/S6 axis. However, this lipid kinase indeed regulates the intrinsic PD-L1 expression in TNBC cells at transcription level, which may be mediated by maintaining the constitutive/intrinsic activation of AKT and then downstream transcription factors. On the other hand, neither IFN-γ nor PMC induced PD-L1 expression is mediated by AKT activation.

### PIPKIγ regulates the transcription of PD-L1 by activating NF-κB

**Figure 4:**
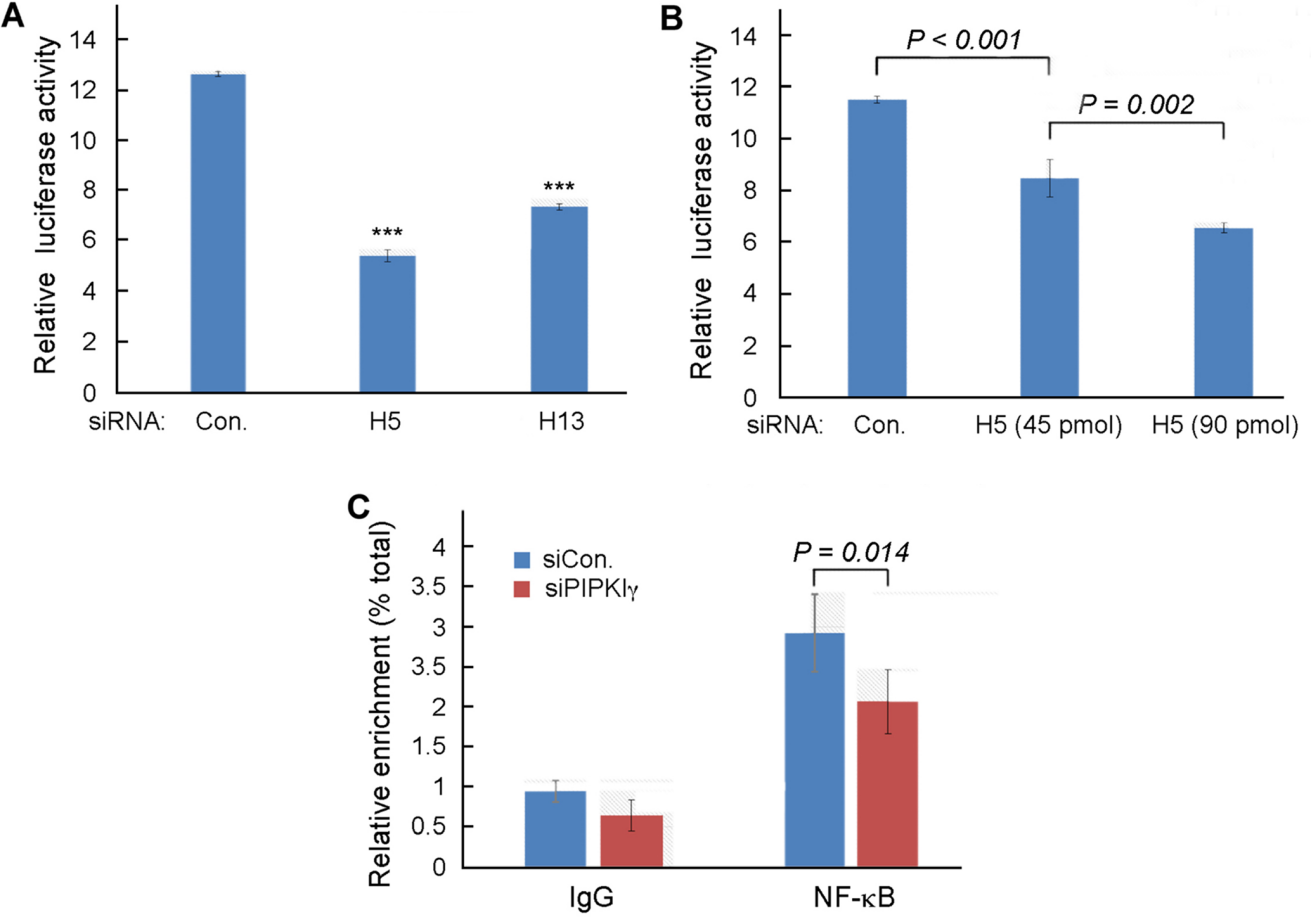
PIPKIγ is required for NF-κB-mediated transcription in TNBC cells (**A**) MDA-MB-231 cells were treated with control or pan-PIPKIγ (H5 or H13) siRNAs for 48 hrs, transfected with control vector pGL3-Basic or NF-κB reporter p1242-3x-κB-L together with pRL-SV40P for 24 hrs, and then subjected to luciferase assay. (**B**) MDA-MB-231 cells were treated with control or indicated amount of pan-PIPKIγ (H5) siRNAs for 48 hrs. These cells were then transfected and analyzed for dual-luciferase assay as described in A. (**C**) Control or PIPKIγ–depleted MDA-MB-231 cells were subjected to ChIP assay using anti-p65 antibody followed by PCR with PD-L1 specific primers. A–C, Results obtained from at least three independent experiments were quantified, statistically analyzed, and then plotted as mean ± s.d.

As described above, our results suggested that the transcription of PD-L1 gene in TNBC cells is regulated by PIPKIγ. Up to date, some transcription factors have been implicated in the intrinsic and/or induced PD-L1 expression in normal and tumor cells. These include AP-1, NF-κB, Jun/Fos, STAT, and HIF-1α, and many of them can be activated by the AKT pathway [33]. To determine which transcription factor may mediate the PIPKIγ-dependent transcription of PD-L1 gene, we firstly examined whose transcription activity shows dependency on PIPKIγ. For this purpose, the luciferase reporter driven by each of these transcription factor-specific promoters was expressed in control or PIPKIγ-depleted HEK 293 cells, then the expression level of luciferase was determined by standard luciferase assay using microplate reader. Among all five specific reporters, the NF-κB reporter was the only one that showed alternated expression when PIPKIγ was knocked down. As summarized in Figure 4A, both PIPKIγ–specific siRNAs caused significant decrease in the activity of NF-κB-driving promoter. Moreover, stronger inhibition of the NF-κB-dependent luciferase expression was observed with higher dose of PIPKIγ-specific siRNA (Figure 4B), further supporting that the transcription activity of NF-κB requires PIPKIγ.

It has been reported that IFN-γ can induce PD-L1 expression by activating NF-κB [36]. In addition, the PKC-activted PKD that induces PD-L1 expression in tumor cells can also activates NF-κB [37, 38]. Thus, our results consistently suggest that PIPKIγ may promotes PD-L1 expression in TNBC cells by regulating NF-κB. To further investigate this possibility, we performed the chromatin immunoprecipitation (ChIP) assay to determine the binding of NF-κB to PD-L1 promoter in TNBC cells with or without depleting PIPKIγ. For this purpose, a pair of PCR primers were designed to amplify the promoter sequence of PD-L1 including 2000-base pair upstream and 100-base pair downstream of the promoter as published at “UCSC Human Gene Sorter” website and further matched at “ gene-regulation.com”. Chromatins prepared from control or PIPKIγ-depleted MDA-MB-231 cells were immunoprecipitated with antibodies specific for the p65 subunit of NF-κB. Results from following qPCR analyses using primers specific for PD-L1 promoter suggested that p65 indeed binds to the promoter of PD-L1 (Figure 4C). More importantly, depletion of PIPKIγ significantly reduced the enrichment of PD-L1 promoter by p65 in comparison with control cells (Figure 4C), indicating that PIPKIγ deficiency impairs the binding of NF-κB to the PD-L1 promoter, which then subsequently inhibits the transcription of PD-L1 gene.

**Figure 5:**
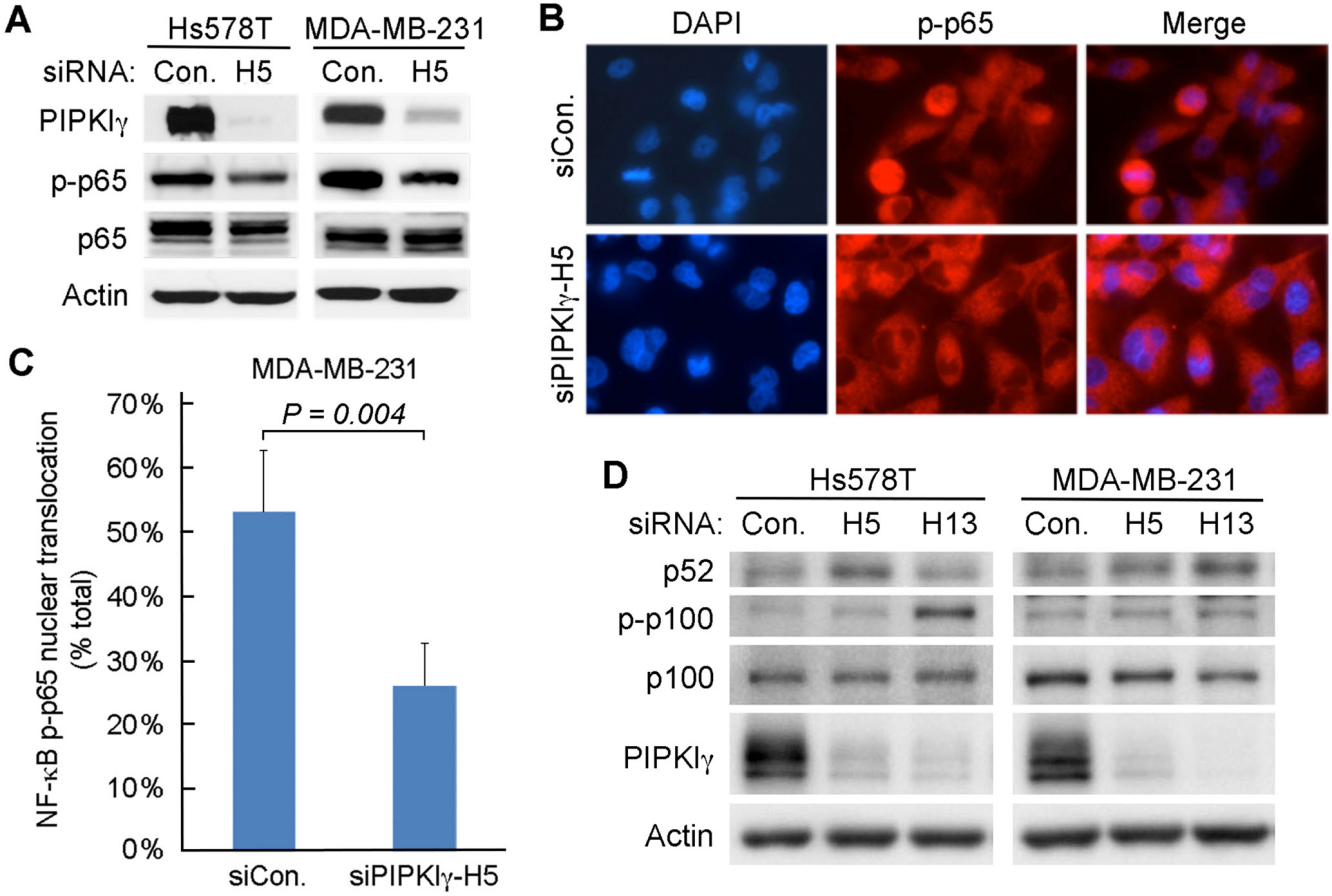
Depletion of PIPKIγ restrains the phosphorylation of p65 (**A**) MDA-MB-231 or Hs578T cells were treated with control (Ctrl) or pan-PIPKIγ (H5) siRNA for 48 hrs, and then analyzed by immunoblotting with indicated antibodies. p-p65, phosphorylated p65. (**B**) MDA-MB-231 cells as described in A were used for indirect immunofluorescence microscopy to determine the nuclear signal of phosphorylated p65. (**C**) Fluorescence intensity of p-p65 in the nucleus were quantified in > 100 cells of each group as described in B, then the results from three independent experiments were statistically analyzed and plotted as mean ± s.d. (**D**) MDA-MB-231 or Hs578T cells were transfected with control (Con.) or two different human pan-PIPKIγ-specific siRNAs (H5 and H13) for 48 hrs. Cells were then lysed and analyzed by Western blot using indicated antibodies. p-p100, Phosphorylated p100.

It has been well established that the nucleus-translocation and phosphorylation of the p65 subunit of NF-κB is essential to the activation of the NF-κB dependent gene expression. To understand how PIPKIγ may regulate NF-κB activity, we first investigated the phosphorylation of serine 536 (pS536) in p65, which plays a critical role in the activation of p65 [39]. As shown in Figure 5A, the levels of pS536-p65 exhibited a significant decrease in both MDA-MB-231 and Hs578T cells upon depletion of PIPKIγ (Figure 5A). Nuclear translocation of pS536-p65, a key step for activating downstream gene transcription [40], also decreased in PIPKIγ-depleted cells in comparison of that in control cells (Figure 5B). Quantification of the nuclear translocation of pS536-p65 (Figure 5C) revealed that it was strongly inhibited in PIPKIγ-depleted cells as compared with the control group (*P* < 0.01). However, total nuclear p65 remained similar in control and PIPKIγ-depleted cells and no change was observed in IκB phosphorylation (data not shown). These results indicate that the destruction of IκB and the following nuclear translocation of p65 are not affected by PIPKIγ deficiency; however, the phosphorylation of p65 at S536 is impaired by PIPKIγ deficiency, which subsequently impaired the transcription of p65-targeted PD-L1 gene. In addition to p65, p52, produced by cleavage of phosphorylated p100 upon activation of conventional NF-κB pathway, also plays an important role in activating the transcription of by forming dimers with p65. However in both MDA-MB-231 and Hs578T cells, neither the phosphorylation level of p100 nor the protein level of p52 decreased in cells treated with PIPKIγ-specific siRNAs comparing to cells treated with control siRNA (Figure 5D). Therefore it seems that PIPKIγ is only required for the S536 phosphorylation of p65.

**Figure 6:**
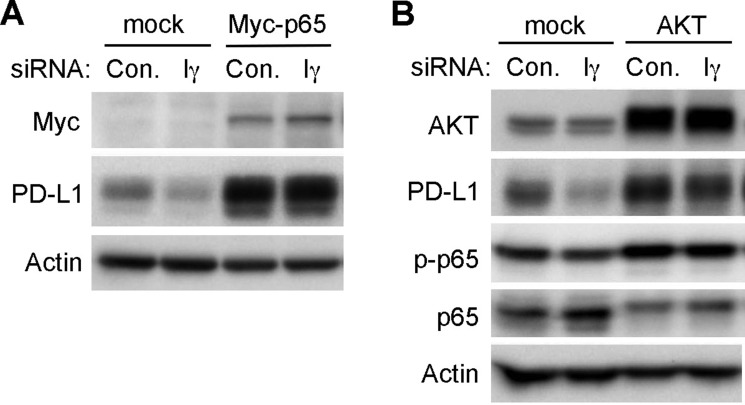
Overexpression of p65 or AKT recovered the downregulation of intrinsic PD-L1 expression caused by PIPKIγ depletion Hs578T cells were transfected with Myc-tagged p65 (**A**) or AKT equally mixed with AKT1, AKT2 and AKT3 constructs (**B**) for 24 hrs, then treated with control (Con.) or human pan-PIPKIγ specific (H5) siRNAs for 48 hrs. Cells were then analyzed by Western blot with indicated antibodies. p-p65, phosphorylated p65.

Many different protein kinases may mediate the phosphorylation of S536 in p65, including IKKs, ribosomal S6 kinase 1 (RSK1), TANK-binding kinase 1 (TBK1) and AKT (Viatour P., Merville, et al., 2005). Since we did not observe any change in the phoshphorylation of IKKα, IKKβ and the ribosomal protein S6 in PIPKIγ-depleted cells, these kinases likely are not the effector mediating PIPKIγ-dependent p65 phosphorylation. However, loss of PIPKIγ indeed weakened the activation of AKT but not the downstream mTOR-mediated S6 protein phosphorylation. Thus, it is quite plausible that the PIPKIγ, by activating AKT and subsequently NF-κB pathway, facilitates the intrinsic PD-L1 expression in TNBC cells. To test this possibility, we first overexpressed Myc-tagged p65 protein in control or PIPKIγ-depleted cells and then checked PD-L1 levels. As shown in Figure 6A, overexpression of p65 significantly increased PD-L1 expression comparing to non-transfected cells, indicating that activation of NF-κB pathway can promote the intrinsic PD-L1 expression in TNBC cells. Furthermore, overexpressed p65 completely recovered the PD-L1 expression in PIPKIγ-depleted cells (Figure 6A), indicating that p65 functions downstream of PIPKIγ in regulating PD-L1 expression. Similarly, upregulation of AKT activity by overexpressing all three AKT isoforms also elevated PD-L1 expression in normal TNBC cells and rescued the PD-L1 downregulation in PIPKIγ-depleted cells. In addition, p65 phosphorylation was increased upon the expression of exogenous AKTs, which also recovered the decreased p65 phosphorylation in PIPKIγ-depleted cells. Together, these results support a model where the PIPKIγ–PI3K–AKT–NF-κB axis plays a major role in regulating the intrinsic PD-L1 expression in TNBC cells. In the context that PIPKIγ depletion has no effect on S6 phosphorylation, another possibility could be that artificially activated AKT by overexpression triggers the induced-PD-L1 expression pathway that is dependent on mTOR-S6K but parallel to PIPKIγ-dependent intrinsic PD-L1 expression.

## DISCUSSION

The PD-1/PD-L1 blockade is a highly valuable strategy that could potentially achieve complete repression and regression-free survival to patients diagnosed with variant types of cancers [41]. However, the low response rate and efficacy indicate that better understanding of the activation and regulation of the PD pathway during cancer progression is necessary. Tumor-associated PD-L1 expression, which may be intrinsically upregulated by activation of tumorigenic pathways or induced by acute (TNF-α and IFN-γ) or continuous/repeated stimuli (chronic inflammation), could inhibit the host immune responses and promote tumor progression. In addition to the critical role played by PD-L1 in tumor immune evasion, recent studies suggest a possible link between PD-L1 induction and malignant conversion. In spite of this, the expression level of PD-L1 is not a reliable biomarker to predict patient response to anti-PD therapies. A recent large-scale study demonstrated that half of all breast cancer expressed PD-L1 mRNA [14, 42]. Although higher PD-L1 mRNA levels are associated closely with elevated tumor-infiltrating lymphocytes (TIL) numbers and longer recurrence-free survival, the majority of breast cancers (46%) exhibits PD-L1 expression but low TIL numbers, who would benefit less from PD-1 blockade therapy alone. In such a group, additional treatments to modulate intracellular signaling associated with cell malignancy and/or to stimulate immune cell recruitment to tumors may be required. Investigation of PD-L1 expression mechanisms will be important to optimize treatment approaches in TNBC patients. Yet, how PD-L1 expression is regulated in TNBC cells is unclear.

Although a recent study indicates that PD-L1 detection in cancer and immune cells varies by antibody clones [43], it has been confirmed that PD-L1 is expressed in at least 20% of the TNBC patients [44]. This study also showed that inhibition of PTEN enhances, whereas inhibition of AKT diminishes, PD-L1 expression in TNBC cells. These results indicate that PD-L1 is regulated by PI3K/AKT pathway in TNBC as in many other types of solid tumors such as non-small cell lung cancer [33, 45]. However, the signaling cascades upstream and transcription factors downstream of PI3K/AKT are unknown. Here we report for the first time that in TNBC cells, both the intrinsic and induced expression of PD-L1 require another lipid kinase called PIPKIγ that functions upstream of PI3K by producing PtdIns(4,5)P_2_, the substrate of PI3K. This regulation is single directional since level of PD-L1 has no effect on PIPKIγ expression. PI3K/AKT and the downstream mTOR/S6 kinase were reported important for activating the translation of PD-L1 protein in variant types of cancers, including non-small cell lung cancer [45], prostate cancer [46], glioma [47], as well as in TNBC [44]. However, loss of PIPKIγ has no effect on the phosphorylation of S6 ribosomal protein in TNBC cells, although it indeed impaired AKT activity. Our results from further investigation demonstrate that PIPKIγ regulates the transcription of PD-L1, instead of influencing the translation of PD-L1. These results define PIPKIγ as a new player in regulating the intrinsic PD-L1 expression in TNBC cells. Up to date, several transcription factors including NF-κB have been implicated in PD-L1 upregulation in different types of cancers [33]. We showed here that PIPKIγ functions upstream of AKT to promote the activation of p65 NF-κB pathway and subsequently PD-L1 transcription.

In addition to regulating the intrinsic PD-L1 expression, we showed that PIPKIγ is also required for IFN-γ and PMA-induced expression of PD-L1. Both IFN-γ and PMA can activate NF-κB-mediated transcription of specific genes such as PD-L1. This is consistent with our current data that NF-κB activity is impaired by loss of PIPKIγ in TNBC cells, therefore leads to inhibition of the constitutive or induced expression of PD-L1 in PIPKIγ-depleted TNBC cells. Instead of inhibiting the nuclear translocation of p65, our data suggest that depletion of PIPKIγ inhibits p65 phosphorylation and binding to the promoter of PD-L1 gene. This is a novel mechanism since PIPKIγ has been known as an important regulator of cytoskeleton reorganization, vesicular trafficking, cell migration and invasion, but not transcription regulation. Although loss of PIPKIγ indeed inhibits the constitutive activation of AKT in un-treated TNBC cells, which is consistent with previously published work [48], IFN-γ or PMA treatment that triggered PD-L1 expression did not induce AKT activation in these cells. However, p65 phosphorylation indeed increased following IFN-γ treatment, which was diminished in PIPKIγ-depleted cells. Although these data support the role of PIPKIγ in regulating the activation of NF-κB downstream of IFN-γ, how PIPKIγ influences p65 phosphorylation remains vague and requires further investigation. Interestingly, NF-κB was implicated in regulating inducible, but not the intrinsic constitutive expression of PD-L1 in melanoma [49]. However in TNBC, our data suggest that NF-κB is required for both intrinsic and inducible expression of PD-L1 in a PIPKIγ-dependent manner.

We previously find that PIPKIγ participates in breast cancer progression and targeting PIPKIγ inhibits the metastasis of breast cancer [30, 31]. Our current results further showed that loss of PIPKIγ suppressed the expression of PD-L1, which likely also contributes to the inhibition of cancer progression caused by PIPKIγ depletion, because loss of PD-L1 expression makes tumor cells much less defensive to anti-tumor immunity. Additionally, recent studies suggested a correlation between PD-L1 and the epithelial-to-mesenchymal transition (EMT) in solid tumors to regulate cancer metastasis [50, 51–54]. The pro-metastatic process EMT may induce breast cancer PD-L1 expression and immune suppression in a PI3K/AKT and/or MEK/ERK dependent manner. PD-L1 expression may also promote and/or maintain the EMT. This positive feedback could lead to the inhibition of the EMT and tumor metastasis when PD-L1 is downregulated by PIPKIγ depletion in TNBC cells [55]. Moreover, it is worth noticing that PIPKIγ-mediated regulation of PD-L1 expression may entangle the EMT-related transcriptional factors. In addition, PI3K/AKT pathway also plays pivotal roles in the EMT and accelerating PD-L1 expression. Thus, there might be complicated crosstalks among the events during the initiation and progression of TNBC.

In summary, we identified PIPKIγ as a novel regulator for both the constitutive and induced PD-L1 expression at the transcriptional level in human TNBC cells. One thing worth noticing is that 4T1 mouse TNBC cells do not have intrinsic PD-L1 expression and the *in vitro* IFN-γ–induced PD-L1 expression does not change when PIPKIγ is knocked down (data not shown). Similarly, the IFN-γ–induced PD-L1 expression in mouse Her2+ breast cancer cells isolated from HER2+ neuT mice is also insensitive to PIPKIγ depletion (data not shown). At this point, we are not sure whether human and mouse breast cancer cells utilize different pathway to regulate PD-L1 expression, or breast cancer cells without intrinsic PD-L1 expression employ signaling cascades different from what utilized by TNBC cells for intrinsic PD-L1 expression. Further work need to be done to sort these possibilities out. Nevertheless, we failed to detect the *in vivo* PD-L1 expression in 4T1 tumors resulted from inoculating 4T1 cells in mouse mammary fat pads (data not shown), although PD-L1 expression indeed can be triggered by IFN-γ in these cells cultured *in vitro*. This suggests that the PIPKIγ-dependent fast progression of 4T1 tumors in mice likely does not rely on PD-L1-dependent immune evasion. In the future, it will be interesting to test whether PIPKIγ-dependent PD-L1 expression may contribute to inhibiting cancer progression using TNBC orthotopic xenotransplantation mouse models (both nude mouse and immune system humanized mouse models), as we reported previously using the 4T1–Balb/C mouse model [31]. Our current results not only shed a light on the molecular mechanisms in human TNBC that promote the constitutive expression of PD-L1 activated by oncogenic pathways, but also PD-L1 upregulation induced by cytokines in the tumor-associated microenvironment including tumor-associated immune cells. Our results and further studies in this direction may suggest alternative strategies to control PD-L1 expression in human TNBC, which may be used to potentiate or sensitize TNBC patients for promising immunotherapies including immune checkpoint blockade.

## MATERIALS AND METHODS

### Cell culture and transfection

Human triple negative breast cancer cell lines (MDA-MB-231, MDA-MB-436, Hs578T) were purchased from ATCC company; PD-L1 knockout MDA-MB-231 cell line created using CRISPR/CAS9 was kindly provided by Dr. Haidong Dong (Mayo clinic, Rochester, MN). All cells were cultured in DMEM supplemented with 10% fetal bovine serum (FBS), 100 U/ml penicillin and 100 mg/ml streptomycin in tissue culture incubator at 37°C with 5% CO_2_.

Stealth RNAi siRNA Negative Control and all siRNA oligonucleotides targeting human PIPKIγ listed below were obtained from Invitrogen.

h5: 5′-GCG TGG TCA AGA TGC ACC TCA AGT T-3′ h6: 5′-CCT ACA GGT TCA TCA AGA AAC TGG A-3′ h12: 5′-CAC CTT TGG GAA GAA TTC CTC TCC A-3′ h13: 5′-CAG GAG GAC GGC AAG ACC TAT TTA T-3′ si668-1 (targeting human PIPKIγ isoform 2): 5′-GAG CGA CAC ATA ATT TCT A-3′

For siRNA transfection, 8 × 10^5^ cells were seeded into 6 well plates and siRNA duplexes were introduced into cells with Lipofectamine RNAiMAX (Invitrogen) according to the manufacturer's manual. Typically 48 hr after transfection, cells were collected for further analysis. RelA construct was a kind gift from Dr. Zhaohui Wu (University of Tennessee). All type I PIPK plasmids were kindly shared by Dr. Richard Anderson (University of Wisconsin-Madison). Mammalian expressing constructs of AKT1, AKT2 and AKT3 were obtained from Addgene. For transient expression of exogenous proteins, 5 × 10^5^ cells were plated in each well of 6-well plates and transfected with XtremeGENE 9 (Roche) following the manufacturer's manual.

### Immunoblotting and Immunofluorescence Microscopy

The proteins were separated using SDS-PAGE and then transferred onto PVDF membranes, blocked with 5% milk for 1 hr. The primary antibodies (1:1000) were incubated overnight at 4°C, followed by incubation with HRP-conjugated secondary antibodies (1:5000) at room temperature for 2 hrs. β-actin (1:10,000) antibody was used as loading control. The membranes were visualized using the Pierce™ Western ECL Blotting substrate (ThermoFisher Scientific, Waltham, MA) and ChemiDoc Touch image system (Bio-Rad).

Cells cultured on glass coverslips were transfected with siRNAs after 48 hrs, then fixed with 4% paraformaldehyde and permeablized with 0.2% Triton X-100 in PBS. After incubating with 3% bovine serum albumin (BSA) in PBS, cells were stained with primary antibodies (1:500 diluted in 3% BSA/PBS) for 1–2 hrs followed by three 5-min washes with 0.1% Triton X-100 in PBS. Then Alexa-conjugated secondary antibodies (1:500 diluted in 3% BSA/PBS) was incubated with the cells in dark for another hour and washed as described above. Cells were then sealed with mounting medium containing DAPI (4′,6-diamidino-2-phenylindole, dihydrochloride), and examined under a Nikon Eclipse fluorescence microscope.

Polyclonal PIPKIγ anti-serum was generated as described [31]. Mouse monoclonal antibody against human PD-L1 was kindly provided by Dr. Haidong Dong (Mayo Clinic). The following antibodies were used for western blot, immunofluorescence and immunohistochemistry: Anti-HA antibody (H6908) and β-actin (A3854) are from Sigma; PD-L1 (#13684), p-S6 (#4858), EMT antibody kit (#9782), NF-κB pathway sampler kit (#9936, #4888), pAKT (Ser473, #4060), AKT (No. 4691), pERK (Thr202/Tyr204, #4370), ERK (#4695), pSTAT3 (Tyr705, #9145), STAT3 (#4904), and phospho-S6 kinase (Thr421/Ser424) antibody (#9202) are from Cell Signaling; Alexa Flour 488 goat anti-mouse antibody and Alexa Flour 555 goat anti-rabbit antibody are from Molecular Probes.

### Quantitative real time-PCR

Breast cancer cells were seeded at a density of 8 × 10^5^ cells/well in 6-well plates and transfected with siRNA. After 48-hr transfection, total RNA was extracted from each well using Qiagen RNeasy kits (Qiagen Valencia, CA). RNA quantity and quality were measured using Nanodrop™ spectrophotometer (NanoDrop products, Wilmington, CA). Complementary DNA (cDNA) was synthesized from 1 μg total RNA by reverse transcription using High Capacity cDNA Reverse Transcription Kit (ThermoFisher Scientific, Waltham, MA). The resulting cDNA was used as template to perform quantitative polymerase chain reaction (qPCR) using CFX384 Touch real-time System (Bio-Rad, Hercules, CA) with iTaq™ Universal SYBR^®^ Green Supermix (Bio-Rad, Hercules, CA). Sequences of gene-specific primers were summarized in Table 1. A 10-μL qPCR reaction mixture contained 2 μl cDNA, 5 μl SYBR Green Supermix, 1 μl forward primer (5 μmol/L), 1 μl reverse primer (5 μmol/L), and 1 μl sterile deionized water. qPCR program is : 95°C × 20 s, 60°C × 20 s, 72°C × 25 s. Melt curve analysis was performed to verify that there was no amplification of nonspecific products with each set of primers. Primers for GAPDH were used to normalize the expression of target genes. Relative quantification was performed using the comparative 2^−ΔΔCt^ method.

**Table 1:** Primer sequence for qRT-PCR and ChIP

Name	Primer sequence
PD-L1 for qRT-PCR	forward 5′-ATA TTC ATG ACC TAC TGG C-3′
	reverse 5′- TAC TAT GCT GAA CCT TCA G-3′
GAPDH	forward 5′-GAA GGT GAA GGT CGG AGT-3′
	reverse 5′-CAT GGG TGG AAT CAT ATT GGA A-3′
PD-L1 for ChIP	forward 5′-CTT CCG CAG CCT TAA TCC TTA-3′
	reverse 5′-ATC GTG GAT TCT GTG ACT TCC TC-3′

### Dual luciferase reporter assay

MDA-MB-231 cells were plated in 6-well plates at 8 × 10^5^ cells/well and transfected with siRNAs. 48-hr after transfection, 2 μg NF-κB promoter construct p1242-3x-κB-L or control vector pGL3-Basic were transfected using Xtreme-Gene9 (Roche) together with 0.02 μg pRL-SV40P, respectively. At 24-hr post-transfection, cells were treated with Dual-Luciferase^®^ Reporter Assay System kit (E1910, Promega, Madison, WI) was used following the manufacturer's instruction. Briefly, 500 μl 1 × PLB were added to cells for 20 min on a rocking platform, and cells were centrifuged at 12000 rpm for 5 min. Supernatants were used for firefly and renilla luciferase activities measurement with Centro XS^3^ LB 960 Microplate Luminometer (Berthold Technology, Germany).

### Chromatin Immunoprecipitation

ChIP assays were performed using a high-sensitivity ChIP Assay kit (Abcam, Cambridge, MA) according to the manufacturer's instructions. Briefly, control or PIPKIγ-depleted cells were fixed with 1% formaldehyde and cross-linked with glycine. After being washed with ice cold PBS, cells were lysed thoroughly in 200 μl lysis buffer for 10 min. After adding 100 μl ChIP buffer, re-suspended chromatin was sheared with Covaris S220 sonicator (Covaris, Woburn, Massachusetts) so that sheared DNA had a peak size of 400 bp, less than 700 bp. Sonicated DNA was used for preparation of ChIP reaction according to the instruction. Anti-p65 antibody (Cat. # 8242, CST, USA) was used to pull down protein/DNA complexes, and the IgG antibody provided by the ChIP kit was used as negative control. The ChIP reaction mixture was incubated at 4°C overnight on the orbital shaker (100 bpm). After washing, reversal cross-linking, releasing and purification of DNA, real-time qPCR was performed with PD-L1 primers (for ChIP as listed above) targeting the endogenous PD-L1 locus to measure the relative enrichment efficiency of different amplicons in the total input ChIP DNA fragments. Results were represented as fold changes relative to the input.

*In vitro* **cell migration assay**

The *in vitro* migration assays were performed in modified Boyden chambers (Neuroprobe, Gaithersburg, MD, USA). The membrane with 8-μm pore was pre-coated with type I collagen (10 μg/ml). The lower compartment was filled with medium containing 10% FBS and serum-starved cells were added to the upper compartment. Cells were allowed to migrate at 37°C for 4–6 hrs. Cells that had not migrated to the other side of the membrane were removed with a cotton swab. Membrane was fixed and stained with DAPI. Migrated cells were quantified by counting 5 high power fields in a blinded fashion.

### Statistical analysis

Results are presented as mean ± s.d. or s.e.m., as specified in each figure legend. Statistical analysis was carried out using one-way ANOVA (SPSS 23.0). *P < 0.05, **P < 0.01 and ***P < 0.001 were considered as statistically significant differences.
